# Draft Genome Sequence of *Larkinella* sp. Strain BK230, Isolated from *Populus deltoides* Roots

**DOI:** 10.1128/MRA.00159-20

**Published:** 2020-03-19

**Authors:** Dale A. Pelletier, Leah H. Burdick, Mircea Podar, Christopher W. Schadt, Udaya C. Kalluri

**Affiliations:** a Biosciences Division, Oak Ridge National Laboratory, Oak Ridge, Tennessee, USA; Georgia Institute of Technology

## Abstract

*Larkinella* sp. strain BK230, a heterotrophic bacterium of the phylum *Bacteroidetes*, was isolated from the roots of a field-grown eastern cottonwood tree (Populus deltoides) located in Georgia. The draft 7.27-Mb genome has a G+C content of 53.4% and contains 6,026 coding sequences, including 41 tRNA genes.

## ANNOUNCEMENT

**FIG 1:**
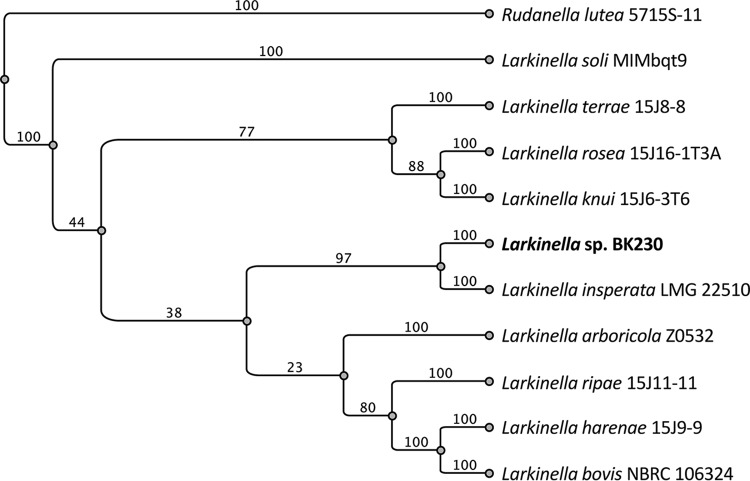
Neighbor-joining phylogenetic tree based on 16S rRNA sequences of *Larkinella* sp. strain BK230 and reference species downloaded from the NCBI database. Rudanella lutea was utilized as an outgroup. The bootstrap support values of the branches are indicated.

The genus *Larkinella*, a member of the *Cytophagaceae* family within the bacterial phylum *Bacteroidetes*, encompasses 9 named species (http://www.bacterio.net/larkinella.html), with only 5 being represented by genome sequence data. Here, we report the draft genome sequence of *Larkinella* sp. strain BK230, which was isolated from a Populus deltoides root sample collected from a field site in Georgia. This genomic information will enable comparative studies of tree root endophytes and the mechanisms of microbe-plant associations. Fine roots (<0.2 mm in diameter) were harvested from *Populus deltoides* WV94, growing on a nursery site in Bellville, Georgia, in September 2017. Washed root tissue was ground, and dilutions were plated on Reasoner’s 2A (R2A) agar and incubated for 7 days at 25°C. Pale-pink colonies that developed were isolated and subsequently identified by small-subunit rRNA gene amplicon sequencing (1). Based on 1,373 bases of 16S sequences from strain BK230 and a number of reference strains, a ClustalW v2.1 alignment and a neighbor-joining phylogenetic tree generated using Geneious v11 (2) indicated that the closest relatives of strain BK230 were Larkinella insperata and Larkinella arboricola, with 96.2% and 95.5% nucleotide identities, respectively (Fig. 1). Therefore, we determined our isolate to be a putative novel species in the genus *Larkinella.* For genome sequencing, *Larkinella* sp. strain BK230 cells were grown in liquid R2A medium overnight at 25°C with shaking. DNA was prepared utilizing a Qiagen DNeasy kit. The draft genome of *Larkinella* sp. strain BK230 was generated at the Department of Energy (DOE) Joint Genome Institute (JGI) using Illumina technology (3). An Illumina standard shotgun library was constructed and sequenced using the Illumina NovaSeq platform, which generated 12,544,362 reads, totaling 1,894,198,662 bp. Raw Illumina sequence data were quality filtered using BBTools (4), according to standard operating procedure 1061. The following steps were then performed for assembly: (i) artifact-filtered and normalized Illumina reads were assembled using SPAdes v3.12.0 (parameters were as follows: phred-offset 33, cov cutoff auto, t 16, m 64, careful, and k 25,55,95) (5); and (ii) contigs were discarded if the length was <1 kbp (BBTools reformat.sh, minlength). The final draft assembly contained 16 contigs in 15 scaffolds, totaling 7,274,818 bp. The final assembly was based on 1,497,902,613 bp of Illumina data, with a mapped coverage of 203.4×. The final genome assembly completeness (100%) and contamination (0.3%) were assessed with CheckM; a metabolic model was generated using KBase (6) and is accessible online (https://narrative.kbase.us/narrative/ws.54100.obj.1). Annotation was performed using the standard IMG Annotation Pipeline v4.16.4, resulting in 6,026 coding sequences, including 5,973 protein-coding genes and 53 RNA genes.

### Data availability.

The complete draft genome sequence is available from the IMG/MER database under accession number 2802428837 and has been deposited in GenBank under accession number SODS00000000. The version described in this paper is the first version (SODS01000000). The raw sequence reads have been deposited in the SRA database under accession number SRP191027.
